# Mandibulofacial Dysostosis Attributed to a Recessive Mutation of *CYP26C1* in Hereford Cattle

**DOI:** 10.3390/genes11111246

**Published:** 2020-10-22

**Authors:** Renae L. Sieck, Anna M. Fuller, Patrick S. Bedwell, Jack A. Ward, Stacy K. Sanders, Shi-Hua Xiang, Sichong Peng, Jessica L. Petersen, David J. Steffen

**Affiliations:** 1 Department of Animal Science, University of Nebraska-Lincoln, Lincoln, NE 68583, USA; renae.sieck@huskers.unl.edu (R.L.S.); anna.fuller@unl.edu (A.M.F.); 2 American Hereford Association, Breed Improvement, Kansas City, MO 64048, USA; sbedwell@hereford.org (P.S.B.); Jward@hereford.org (J.A.W.); ssanders@hereford.org (S.K.S.); 3 School of Veterinary Medicine and Biomedical Sciences, University of Nebraska-Lincoln, Lincoln, NE 68583, USA; sxiang2@unl.edu (S.-H.X.); dsteffen1@unl.edu (D.J.S.); 4 Department of Population Health and Reproduction, University of California-Davis, Davis, CA 95616, USA; scpeng@ucdavis.edu

**Keywords:** congenital defect, retinoic acid signaling, animal models, first pharyngeal arch, *Bos taurus*, de novo mutation

## Abstract

In spring 2020, six Hereford calves presented with congenital facial deformities attributed to a condition we termed mandibulofacial dysostosis (MD). Affected calves shared hallmark features of a variably shortened and/or asymmetric lower mandible and bilateral skin tags present 2–10 cm caudal to the commissure of the lips. Pedigree analysis revealed a single common ancestor shared by the sire and dam of each affected calf. Whole-genome sequencing (WGS) of 20 animals led to the discovery of a variant (Chr26 g. 14404993T>C) in Exon 3 of *CYP26C1* associated with MD. This missense mutation (p.L188P), is located in an α helix of the protein, which the identified amino acid substitution is predicted to break. The implication of this mutation was further validated through genotyping 2 additional affected calves, 760 other Herefords, and by evaluation of available WGS data from over 2500 other individuals. Only the affected individuals were homozygous for the variant and all heterozygotes had at least one pedigree tie to the suspect founder. *CYP26C1* plays a vital role in tissue-specific regulation of retinoic acid (RA) during embryonic development. Dysregulation of RA can result in teratogenesis by altering the endothelin-1 signaling pathway affecting the expression of *Dlx* genes, critical to mandibulofacial development. We postulate that this recessive missense mutation in *CYP26C1* impacts the catalytic activity of the encoded enzyme, leading to excess RA resulting in the observed MD phenotype.

## 1. Introduction

Over 250 Mendelian traits in cattle are reported in the Online Mendelian Inheritance in Animals database (https://omia.org/home). Often deleterious syndromes in cattle are attributed to variants inherited in an autosomal recessive manner [1]. Due to this inheritance pattern, clinical signs of disease may not appear for many generations after the causal mutation originates. However, artificial selection in livestock and the commonplace use of artificial insemination and embryo transfer can expedite widespread proliferation of a deleterious variant. Once a deleterious defect is identified, prompt identification of carrier animals is necessary to prevent economic loss. The significant impact of a single deleterious variant in livestock can be exemplified by a recessively inherited mutation in *APAF1*, traced back to a Holstein bull born in 1962. The recessive genotype, detrimental to cow fertility, was estimated to have resulted in 525,000 abortions costing the industry $420 million [2].

In March and April of 2020, three herds reported a total of six purebred Hereford calves born with unusual defects of the face and jaw attributed to a condition we termed mandibulofacial dysostosis (MD). One of the reporting herds also noted a calf born with a similar phenotype in 2018. The calves were live born, of normal size, and with an intact suckle reflex but weak suckling ability. Grossly, several calves appeared to have a widened upturned “smile,” a variably shortened and/or asymmetric lower mandible, and unique, bilateral skin tags just caudal to the commissure of the lips. The three herds reporting MD cases were each in a different state (Iowa, Wyoming, Missouri) with typical summer grazing and winter feeding programs, making an environmental cause unlikely. Sires and dams of affected calves were consanguineous.

The similarity in the reported clinical description among the affected calves, pedigree analysis, and description of similar phenotypes in children [3] and mice [4,5] led to the hypothesis that a de novo, autosomal recessive mutation may be causative of this novel condition in Hereford cattle. Pathologic findings of retained Meckel’s cartilage in the affected calves further suggested such a mutation might disrupt development of the first pharyngeal arch (PA1), possibly through endothelin-1 (ET1) and Dlx signaling. Expression of *Dlx* homeobox genes in the cranial neural crest cells (CNCC) of the embryonic PA1 provides patterning information during jaw formation [4,6]. More specifically, ET1 signaling in PA1 activates *Dlx5* and *Dlx6*, driving differentiation of the mandible and maxilla [5,6]. The identification of a causative variant would allow breeders to identify carrier individuals to avoid production of affected calves; it also has the potential to yield novel information regarding the regulation of craniofacial development. To investigate our hypothesis, multiple affected calves were obtained to establish a phenotypic characterization of the defect and case definition. DNA from these calves, family members, and herd mates was collected for whole-genome sequencing (WGS) to identify possible de novo variation impacting mandibulofacial development.

## 2. Materials and Methods

Animals utilized in this study were sampled in compliance with approved University of Nebraska IACUC project number 1970: Diagnostic Investigation into Natural Animal Disease Events. Five affected calves, two heifers and three bulls, were received at the University of Nebraska-Lincoln Veterinary Diagnostic Center (UNL-VDC) for autopsy evaluation, four after euthanasia on the farm and one live, within 24 h of birth. The live calf was evaluated for hearing and vision and then euthanized by intravenous injection of pentobarbital sodium and phenytoin sodium (Euthasol, Virbac AH, Inc., Fort Worth, TX, USA).

Sire and dam of all reported, affected calves as well as their extended pedigrees (records available through the American Hereford Association, https://hereford.org) were evaluated to identify common ancestors. The herd of origin and date of birth was also noted.

Tissue samples (ear) were collected from the 5 affected calves received at the UNL-VDC. Semen or whole blood samples were obtained from parents of affected calves (as available) and initially from 7 other related individuals. All samples were stored at −20 °C. DNA was isolated from EDTA blood samples with a Gentra Puregene Blood Kit (Qiagen, Venlo, Netherlands) utilizing the following modified protocol. To obtain the buffy coat, blood tubes were centrifuged at 2000× *g* (15 min, 4 °C). 250 µL of buffy coat was combined with 900 µL red blood cell lysis solution, vortexed, and incubated (5 min, 22 °C). Samples were then centrifuged (13,000× *g*, 2 min) and the supernatant discarded. 450 µL of red blood cell lysis solution was added to the pellet, vortexed, and incubated (5 min, 22 °C). Samples were again centrifuged (13,000× *g*, 2 min) and supernatant discarded before adding 900 µL cell lysis solution and 6 µL Proteinase K. The samples were vortexed then incubated (30 min, 35 °C). After cooling on ice to room temperature, 200 µL of protein precipitation solution was added, the samples vortexed, and incubated on ice (5 min). The tubes were centrifuged (13,000× *g*, 2 min). To precipitate DNA, the supernatant was poured into a new tube containing 800 µL of 100% isopropanol, the tubes were inverted 50 times, and centrifuged (8000× *g*, 2 min). The supernatant was discarded and pellet dried for 1 min before washing with 300 µL of 70 % ethanol. The cleaned pellet was dried for 15 min before the DNA was rehydrated in 100 µL of DNA hydration solution overnight (22 °C) before storage at 4 °C.

DNA isolation from semen was also completed using the Gentra Puregene Blood Kit (Qiagen, Venlo, Netherlands) with modifications as previously described [7]. DNA quality and purity were evaluated with an Epoch Microplate Reader (BioTek, Winooski, VT, USA). Isolated DNA samples from 20 individuals underwent KAPA library preparation and sequencing with 150-bp paired-end reads across one lane of an Illumina NovaSeq S4 at Admera Health (South Plainfield, NJ, USA). Adapters and poor-quality bases (minimum Phred score 20) were removed using Cutadapt [8] via the wrapper TrimGalore version 0.4 (https://github.com/Felix Krueger/TrimGalore). The sequences were mapped to the ARS-UCD1.2 reference genome using BWA–MEM [9] and the output .sam files converted to .bam files and indexed using SAMtools [10]. Duplicate reads were marked using Picard (http://broadinstitute.github.io/picard). Variants were called across all individuals with freebayes-parallel (https://github.com/ekg/freebayes/blob/master/scripts/freebayes-parallel), and those with a quality score lower than 30 eliminated. Variant positions (e.g., intronic, exonic) were annotated using ARS-UCD1.2 Annotation Release 106.

Filtering to identify candidate variants was completed utilizing VCFtools (https://vcftools.github.io). Candidate variants were identified as those homozygous for the alternative allele in all affected calves, found in a heterozygous state in all obligate carriers (parents of affected calves), and either heterozygous or homozygous for the reference allele in the related individuals. Variants were further prioritized by evaluating their predicted impact on gene/protein function as annotated by the Ensembl Variant Effect Predictor (https://uswest.ensembl.org/info/docs/tools/vep/index.html) and SNPEff [11].

Sequence Read Archive (SRA) data were obtained for animals mapped to the ARS-UCD1.2 and UMD3.1 reference genomes using a workflow based upon https://github.com/SichongP/SRA_variant_search. The NCBI Genome Remapping Service (https://www.ncbi.nlm.nih.gov/genome/tools/remap) was used to translate variant positions between genome builds. The pileup function from sra-tools (https://github.com/ncbi/sra-tools, version 2.9.1) was used to generate pileup files at candidate variant loci. Subsequent genotyping was carried out for individuals with at least 10 reads per locus at the candidate loci.

For verification of the leading candidate SNP, 762 additional Hereford samples (semen, EDTA blood, or hair) were obtained. Of these samples, 289 were banked DNA samples from the American Hereford Association stored at Neogen GeneSeek (Lincoln, NE, USA), which were received in the form of isolated DNA. DNA was isolated from the blood and semen samples as described above. DNA from hair was isolated as previously described [12].

One of three genotyping methods was employed for validation of the candidate variant. Kompetitive Allele Specific PCR (KASP) genotyping was conducted using primers and probes designed with the KASP on Demand utility (LGC Genomics, Teddington, Middlesex, UK; Table S1). All KASP reactions were performed in duplicate on a CFX384 Touch Real-Time PCR machine (Bio-Rad Laboratories, Hercules, CA, USA) following the manufacturer’s protocol. Non-template (negative) controls, three homozygous reference controls, three heterozygous controls, and four homozygous variant controls were run on each plate. The fluorophores HEX and FAM labeled the wildtype and variant probes, respectively. Results were visualized in CFX Maestro Software (Bio-Rad Laboratories, Hercules, CA, USA).

Any sample failing to genotype in duplicate via KASP was genotyped by either Sanger sequencing or droplet digital PCR (ddPCR). For ddPCR assays, primers to amplify a 136-bp fragment containing the candidate variant were designed in Primer3 [13] (Table S1). PrimeTime, double-quenched (ZEN/IowaBlack FQ) competitive probes were constructed to distinguish between the wildtype (T) and variant (C) alleles, labeled with HEX and FAM fluorophores, respectively. ddPCR reactions were performed using standard protocol on a QX200 ddPCR system (Bio-Rad Laboratories, Hercules, CA, USA). A non-template (negative) control was included as well as positive controls, which consisted of DNA from two affected calves, two obligate carriers, and two unaffected individuals. Samples of interest for allele quantification were run in duplicate or triplicate. The reaction included 1X ddPCR Supermix for Probes (no dUTP; Bio-Rad Laboratories, Hercules, CA, USA), 25 ng (10 ng/µL) of template DNA, primers at 0.18 µM each, probes at 0.02 µM each, and molecular grade water to a final reaction volume of 22 µL. Reactions were converted into approximately 20,000 one-nanoliter droplets using the QX200 Droplet Generator. Thermocycling included 10 min of enzyme activation at 95 °C and 39 cycles of denaturation (94 °C, 30 s) followed by annealing/extension (64 °C, 1 min). Enzyme deactivation (98 °C, 10 min) concluded the cycle. The plate was read in the QX100 Droplet Reader (Bio-Rad Laboratories, Hercules, CA, USA) and results were analyzed using QuantaSoft Software (Bio-Rad Laboratories, Hercules, CA, USA). Power to distinguish alleles was determined from the false-negative rate of the controls (e.g., power for detection of a variant allele = 1 − (wildtype droplets/total positive droplets), when template representing only the variant allele was provided).

Sanger sequencing was performed for 6 different variants at ACGT, Inc. (Wheeling, IL, USA) after PCR with primers designed in Primer3 [13] (Table S1). PCR reactions were performed using a FastStart kit (Sigma-Aldrich, St. Louis, MO, USA) and included 4.45 µL water, 0.25 µL MgCl, 1.2 µL 10X buffer with MgCl, 0.5 µL dNTP, 0.1 µL Taq, 0.75 µL of 20 µM forward and reverse primer, and 4 µL of 5 ng/µL DNA template. Thermal cycling conditions consisted of 94 °C for 4 min, 32 cycles of 94 °C for 30 s, annealing temperature (Table S1) for 30 s, 72 °C for 45 s, a final extension at 72 °C for 10 min, then a 10 °C hold. PCR product cleanup was performed using 0.75 µL ExoSAP-IT (Applied Biosystems, Foster City, CA, USA) per 4 µL PCR product. Thermal cycling conditions consisted of a cycle at 37 °C for 30 min, 80 °C for 15 min, and 15 °C hold.

The cytochrome CYP26C1 (*Bos taurus*) structural model was generated based on the X-ray crystal structure of cyanobacterial CYP120A1 [14] (PDB: 2VE3) using sequence homology modeling program Modeller [15]; their protein sequences have a 31.4% identify and 46.8% similarity. The secondary structural predictions were conducted using Jnet online with the window size setup for 22 amino acids based on the structure of the α-helix segment from 179 to 200 amino acids. Conservation of the amino acid altered by the candidate SNP was also evaluated across multiple species using Multialign (http://multalin.toulouse.inra.fr/multalin).

Whole-genome sequence data generated for this project are available on the NCBI SRA (BioProject ID: PRJNA663547). Sanger sequencing data representing each *CYP26C1* genotype have been deposited in GenBank (Accessions: MW123048 and MW123049). Novel candidate variants have been deposited in the European Variation Archive (EVA) (Project ID: PRJEB40605).

## 3. Results

### 3.1. Pathologic Characteristics of Affected Calves

**Figure 1:**
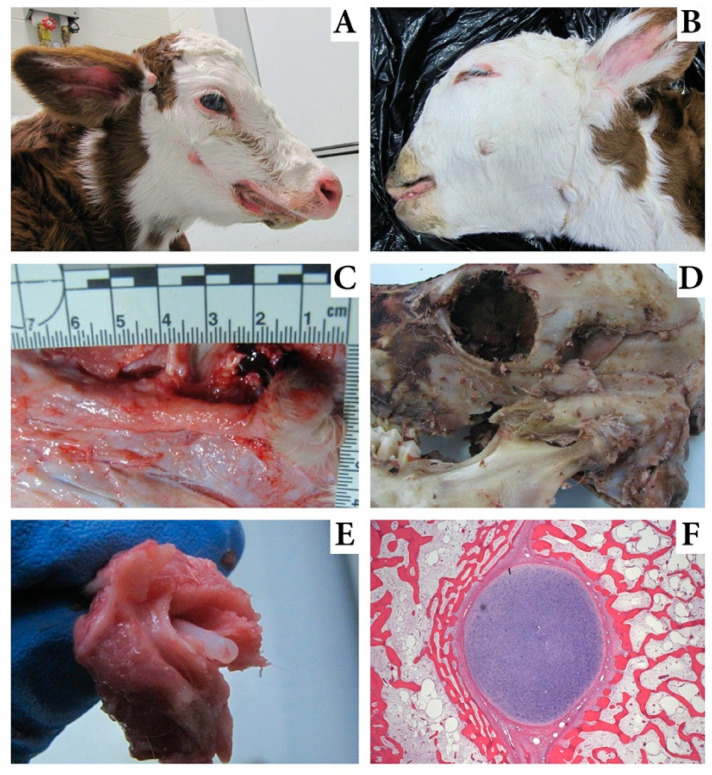
Images of calves affected with mandibulofacial dysostosis (MD). (**A**). An MD calf with megastomia. Skin tags are visible ventral to the eye and at the base of the ear. Brachygnathia is also evident and a slight facial bulge is seen dorsal and caudal to the skin tag. (**B**). An MD calf with skin tags; one is caudal to commissure of the lips and one is ventral to the base of the ear near the caudal ramus of the mandible. (**C**). Exposure of the abnormal bone in an MD calf with the skin tag intact at the right margin. (**D**). The skull of an MD calf showing the exposed bone fractured during autopsy and demonstrating origin of this abnormal bone just above the temporal mandibular joint. (**E**). An image of the fractured bony prominence in an MD calf exposing the retained Meckel’s cartilage within the bony prominence. (**F**). Histology evaluation of the Meckel’s cartilage core from an MD calf surrounded by bone and separated by fibrous tissue.

At autopsy, the 5 MD calves weighed between 32 and 41 kg. Unique and consistent hallmarks of the condition included bilateral skin tags 2–10 cm caudal to the commissure of the lips (Figure 1A,B). The tags were 0.5–2.0 cm long with a central dermal core that attached through a short, 1 cm dermal band to cartilage (Figure 1C). This cartilage extended to its origin at the zygomatic process of the temporal bone (Figure 1D). The cartilage, from its origin, extended cranially for 3–5 cm and was encased in bone. The bony processes were 1.5–2.0 cm wide, 1.0 cm thick, and separated from underlying bones of the face by a 0.5 cm gap. A short, 1.0 cm dorsal lateral protrusion of bone at the origin of the bony process was also present. The bone-wrapped Meckel’s cartilage was bilateral and consistent in each affected calf. When the bone was fractured during autopsy, a perfectly round 0.35 mm diameter tube of cartilage freely separated from the center of the bone (Figure 1E). Several affected calves had additional skin tags near or several centimeters below the external acoustic meatus not associated with cartilage or bone (Figure 1A,B). Additional coexisting facial deformities included megastomia in three, camplyognathia of the mandible in two, campylognathia involving mandible and maxilla in one, and brachgnathia inferior in three calves (Table 1). A single calf had a cleft palate. The calves with maxillary campylognathia also had asymmetry of the orbits with one located approximately 3 cm caudal to the other. All calves had hypoplasia of the masseter and temporalis muscles and pinnae that were low set and drooped.

Histologic evaluation of the bony process originating from the temporal bone revealed a cartilage (Meckel’s) core sandwiched between plates of bone (Figure 1F). The bone and cartilage were surrounded by thick fibrous periosteum. No evidence of endochondral ossification of this cartilage was noted. Sections of eye, kidney, liver, brain, adrenal, spleen, skeletal muscle, thymus, intestine, and lymph node appeared normal. The skin tag had a thick dermal core with redundant collagenous stroma and most sections included normal adnexa and sinus hairs.

**Figure 2:**
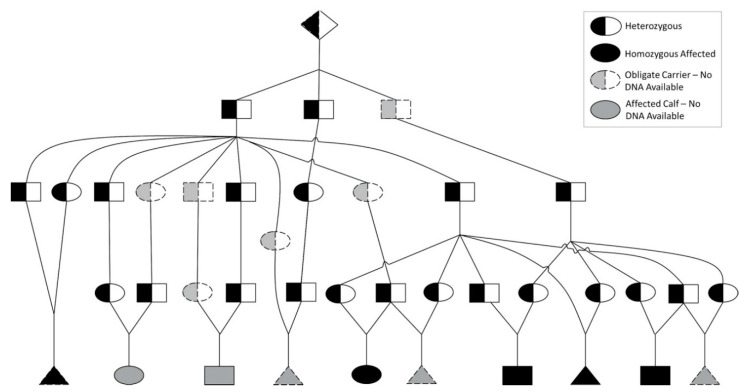
Pedigree of Affected Animals. Pedigree of all MD affected calves (*N* = 10) including those in the WGS dataset and those reported after disclosure of the MD defect to the breed association membership (males = rectangles, females = ovals, unknown sex = triangles, presumed founder = diamond). Animals for which DNA was not available are shown in grey; all others (black) were genotyped for the *CYP26C1* variant.

After disclosure of the MD defect to the breed association membership, three additional affected calves were reported, with the phenotype of two confirmed via digital image evaluation, and one by evaluation at the UNL-VDC. All affected calves had the presumed founder in their pedigree (Figure 2).

### 3.2. Whole-Genome Sequencing

Whole-genome sequence data averaged 13.0X coverage (range 10.8 to 15.5; standard deviation 1.32) and included 20 individuals (3 affected calves, parents of the sentinel cases (*N* = 6), parents of other affected calves (*N* = 4; including that of the 2018 calf) and 7 related individuals) (Figure S1). 143 variants matched the hypothesized recessive mode of inheritance; all but 5 had RefSNP (rs) identifiers. 134 of the candidate variants were located between 10.3 and 15.9 Mb on chromosome 26 (Table S2). Evaluating the variants homozygous in the three affected calves without regard to the genotype of other individuals identified an extended region of homozygosity that also included this region of chromosome 26 (Table S3).

Additional evaluation was performed using WGS data from 101 animals sequenced for other projects in the lab (including 6 purebred Hereford and 8 Hereford-crosses), from 1577 cattle sequenced previously as part of the 1000 Bull Genomes Project [16], and 128 other Herefords provided by the American Hereford Association. These data resulted in the elimination of 141 of the 149 candidate variants as one or more of the additional animals were homozygous for the alternative allele. Of the remaining eight variants, one was a missense variant in Exon 3 of *CYP26C1* (Chr26 g. 14404993T>C; ss7148511443), one SNP was located in the 5′ untranslated region of *TBC1D12* (Chr26 g. 15898152C>T; ss7148511444)*,* and the other 5 were intronic or intergenic (Table 2). The missense variant was the only variant with a predicted impact (SIFT score = 0, deleterious).

### 3.3. Candidate Variant Filtering

A query of the SRA yielded results for 3191 cattle at one or more of the 8 variants queried. These samples represented Hereford, Angus, Red Angus, and Simmental, among other breeds. At 6 of the remaining 8 variants queried, all cattle in the SRA search were homozygous reference, providing no information from which to further filter them from the data set. Two variants were eliminated as possible causative variants due to their presence in other breeds as MD appears to be unique to the Hereford and associated with one specific bloodline. For the first, Chr26 g. 10713132G>A, 6 of 1018 samples with genotype data were heterozygous for the alternative allele (frequency: 0.006). These 6 samples were of breeds including Romagnola, Simmental, and Original Braunvieh. At Chr26 g. 10794674G>A, 7 of 1083 samples were heterozygous and 1 homozygous for the alternative allele (frequency: 0.0083). These 8 samples were also Romagnola, Simmental, and Original Braunvieh.

Sanger sequencing was performed on the 6 remaining candidate variants in one of the additional affected calves, three additional parents of affected calves, the presumed founder bull, the sire of the presumed founder, and 25 animals with the presumed founder in both their maternal and paternal pedigree. These data ruled out 4 of the 6 remaining candidate variants due to identification of animals that genotyped homozygous for the alternative allele but did not display the MD phenotype or due to the MD affected calf not genotyping as homozygous for the alternative allele. Of the two variants that could not be ruled out with additional data (Chr26 g. 14404993T>C and Chr26 g. 15898152C>T), due to its predicted function, the missense variant in *CYP26C1* was the primary candidate of interest and focused upon for further analyses.

### 3.4. CYP26C1 Variant Genotyping

782 Hereford cattle were genotyped for the *CYP26C1* SNP including the WGS animals (*N* = 20), other cattle from two of the reporting herds (*N* = 623), the suspect founder, two additional affected calves, and parents of those affected calves (*N* = 4) (Table 3). Of these 782, 624 were homozygous for the reference allele, 153 heterozygous, and the 5 affected calves homozygous for the variant allele. Within these Herefords genotyped, 106 had the presumed founder bull in both the sire and dam side of their pedigree, 327 had the presumed founder bull in one side of their pedigree, and 348 animals were not descendants of the presumed founder (Table 3). Notably, the suspect founder genotyped heterozygous, all additional affected calves from which DNA was available were homozygous for the alternative allele, and parents of the additional affected calves were heterozygous (DNA on one dam not available). Twelve animals were calves of both a sire and dam that were confirmed carriers of the *CYP26C1* variant allele; of these, 2 were homozygous for the reference allele, 5 heterozygous, and 5 homozygous for the variant allele (all affected with the described MD phenotype).

In total, 3371 genotypes for the *CYP26C1* g. 14404993T>C locus were examined (Table 4). With the exception of 17 of the 20 cattle sequenced for this study, the variant was not identified in any WGS-derived genotype.

Power to distinguish between wildtype and variant alleles by ddPCR was 0.999 and 0.998, respectively. An average of 9999.9 droplets (st dev = 1599.8) were read per sample, with each run in 2 or more replicates. The germ line variant allele frequency of the maternal grandsire and great grandsire of the suspect founder did not exceed that of wildtype controls (Table S4), refuting a hypothesized mosaic origin of the variant in either bull.

### 3.5. Predicted Impact on Protein Function

**Figure 3:**
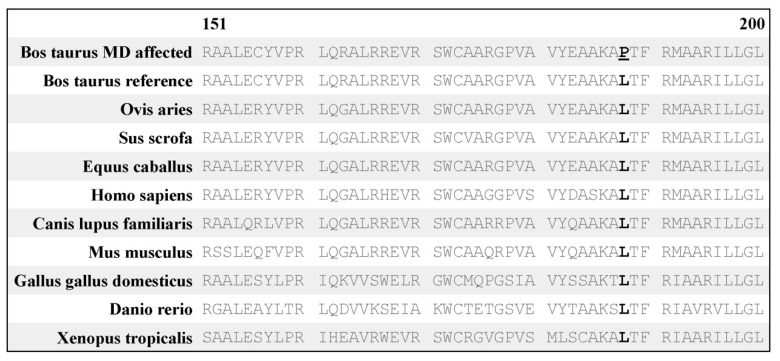
Conservation of the CYP26C1 protein across species. Shown is a portion of the CYP26C1 protein (amino acids 151 to 200 of 523 in the ARS-UCD1.2 reference assembly). Amino acid 188 (bold), altered in mandibulofacial dysostosis calves, is conserved across all other species studied.

**Figure 4:**
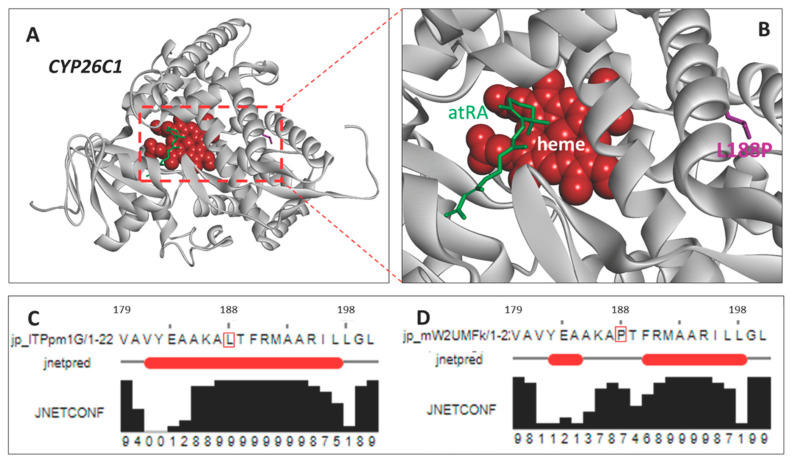
Structural modeling of cytochrome CYP26C1. (**A**). Three dimensional structural model of CYP26C1 (*B. taurus*) showing the heme in red ball, all trans-retinoic acid (atRA) in green stick and the L188P mutation in magenta stick. (**B**). The active site focused structure. (**C**). The secondary structure prediction of the wild-type L188 segment (positions 179–200). (**D**). The secondary structure prediction of the 179–200 segment with the L188P mutation. In (**C**,**D**), the red bars (jnetpred) indicate the predicted position of a helix, which is broken in the presence of the L188P mutation. Confidence in the predicted structure (JNETCONF) is displayed by vertical black bars.

Alignment of the amino acid sequence of CYP26C1 indicated that the residue altered (p. L188P) was conserved across *B. taurus*, *Ovis aries*, *Sus scrofa*, *Equus caballus*, *Homo sapiens*, *Mus musculus*, *Danio rerio*, *Xenopus tropicalis*, and *Canis lupus familiaris* (Figure 3). Structural modeling demonstrated the L188P mutation site is located in an α-helix segment from 179 to 200 amino acids. This is not within the heme active site at which the substrate, all-trans-retinoic acid (atRA), binds. Secondary structural predictions, however, indicate the α-helix will be broken if leucine (L) is substituted with proline (P) at position 188 (Figure 4).

## 4. Discussion

In this investigation, MD of Hereford calves was associated with a missense mutation in *CYP26C1*. MD is detrimental to the viability of affected animals due to an impaired ability to nurse. The identification of this associated and putatively causative marker provides breeders the ability to test for carriers to avoid mating decisions that may produce an affected calf. Animals with the presumed founder on both sides of their pedigree originated from over 40 different breeders showing the widespread implications of MD within the Hereford breed. Further, the implication of *CYP26C1* provides insight into the role of this gene in the regulation of RA during the development of craniofacial structures.

Facial structures including the mandible, maxillary musculature, and some structures of the inner ear and hindbrain are derived embryonically from CNCC that migrate from the neural tube to PA1. This region of the embryo is the most rostral of the anteroposteriorally segmented structures present in early development [17]. Expression of *Dlx* homeobox genes in the CNCC of PA1 provides patterning information that results in specification of the mandibular and maxillary arches [4,6]. Differentiation between the mandible and maxilla is driven by ET1 signaling in PA1 that activates *Dlx5* and *Dlx6* resulting in formation of Meckel’s cartilage in the mandibular, but not the maxillary prominence [5,6]; Meckel’s cartilage, a transient cartilaginous plate, serves as a scaffold during formation and elongation of the intramembranous mandible bone [18].

Migration and proliferation of CNCC is guided by gradients of signals including fibroblast growth factor, retinoic acid, and Wnt [6,19]. Retinoic acid (RA) plays a critical role in PA1 development because it acts as a repressor of ET1 [5]. Excess RA disrupts ET1 signaling acting on the *Dlx* genes. This excess exposure of PA1 to RA results in teratogenesis of the mandible, specifically altering development of the Meckel’s cartilage [5]. Similar effects were observed in both Dlx5/Dlx6 and ET1 receptor mutant mice [20], supporting the importance of RA in the development of mandibular structures. Dysregulation of the RA gradient has the most severe effect on craniofacial morphology at the 9 to 14 somite stage of development [5]; during this period, migrating CNCC reach PA1 [21]. Nine-somite mouse embryos treated with excess RA had malformed Meckel’s cartilage [4,5] while the cartilage developed normally when treated outside of this developmental time point [5]. For cattle, this critical developmental window would fall around day 21 or 22 of gestation [22,23].

The concentration of RA in the developing embryo is regulated in a tissue-specific manner by opposing actions of synthesizing (Raldh) and metabolizing (Cyp26) enzymes [24]. The *CYP26C1* gene and its counterparts, *CYP26A1* and *CYP26B1*, oxidize RA into inactive polar metabolites enabling the maintenance of the RA gradient across the pharyngeal arches [25]. Studies elucidating the expression patterns of *CYP26C1* show it is expressed in PA1 during the critical time point for RA teratogenesis [24].

There are multiple lines of evidence that alteration of *CYP26C1* function results in developmental abnormalities. Knockdown of *CYP26C1* in zebrafish embryos resulted in increased RA levels associated with atypical development of the pharyngeal arches and otic vesicles [26]. *CYP26A1* and *CYP26C1* null mice also had deficient development of PA1 and PA2 and altered migration of CNCC [27]. These studies indicate that normal function of *CYP26C1* is critical to avoid morphological changes in craniofacial structures.

We postulate that the association of the *CYP26C1* missense mutation with abnormal development of the jaw in MD calves can be attributed to the loss of catalytic activity resulting in reduced ability of the enzyme to metabolize RA. The affected amino acid is not located in the canonical heme thiolate-binding motif (FxxGxxxCxG) that is characteristic of the P450 family of proteins to which CYP26C1 belongs [28]. However, Kim and Kang [29] showed proline is a helix breaker and our model supports that the L188P mutation results in a structural change of the α-helix. This change may affect the active site structure for substrate binding and cause a subsequent loss of function impairing metabolism of RA. Further, this amino acid residue is conserved across species. Both the protein model and the evolutionary maintenance of the amino acid serve as evidence the *CYP26C1* L188P mutation is deleterious to protein function.

Reports of craniofacial deformities in cattle commonly include cleft palate or more severe dysplasias [30,31,32]. A recent report from India identified a calf with a pathology similar to MD [33]. This, however, is the first comprehensive report of the novel MD phenotype in Herefords, which we believe is attributed to a de novo mutation in the sire common to the paternal and maternal pedigrees of all affected calves. Semen from the suspect founder genotyped heterozygous for the *CYP26C1* SNP with no evidence of germ-line mosaicism as studied by ddPCR. Semen of this bulls’ sire and both maternal grandsires were homozygous for the reference allele at the *CYP26C1* SNP, also confirmed by ddPCR. DNA was not available from the (deceased) mother of the presumed founder bull, therefore origination of the variant in the maternal lineage of the presumed founder cannot be excluded. Even with the possibility of maternal origin, the bull common to all pedigrees of affected calves has been the primary perpetuator of the variant resulting in the manifestation of this phenotype. To this point, no carrier animals have been identified that do not have the presumed founder in their pedigree. Further, both the presumed founder and his sire genotyped as heterozygous for the only other remaining candidate variant (Chr26 g. 15898152C>T). If that variant was instead causative, we would expect to have identified MD cases in descendants of the sire of the suspect founder without a pedigree connection to the suspect founder himself; that has not been the case, contributing to the body of evidence that the variant in *CYP26C1* is causative of this condition.

Several human conditions exhibit similar pathologies to MD calves. One hallmark of MD in all affected calves is the presence of skin tags located along the fusion site of the maxillary and mandibular prominences. Strikingly similar skin tags are observed in Hemifacial Microsomia patients [3]. Human focal facial dermal dysplasia, Type IV, is also characterized by skin lesions and polyps on the buccal mucosa located at the same fusion site [28]. Notably, both human syndromes are attributed to similar developmental pathways as implicated in MD. Focal facial dermal dysplasia is caused by loss of function mutations in *CYP26C1* [28] and one model of causation for hemifacial microsomia includes altered migration, differentiation, and proliferation of CNCC in PA1 [3].

A condition also termed mandibulofacial dysostosis is described in human literature as a heterogeneous anatomic group of disorders; the described condition in Hereford cattle is not analogous to human MD. The human MD condition more broadly affects chondrocyte and osteoblast differentiation with dysostosis most apparent in, but not limited to, the face [34]. Implicated mutations in *EFTUD2* in human MD are unrelated to CNCC migration and differentiation [35,36]. Further, the normal facial features of humans and bovines differ dramatically with cattle being more dolichocephalic; thus, we do not anticipate homologous anatomic outcomes due to disruptions in branchial arch related processes.

A defining and novel characteristic of Hereford MD is the retention of Meckel’s cartilage past early development and shortening of the mandible. No human conditions have been described exhibiting this precise pathology, but similarities in a group of human syndromes termed “retinoic acid embryopathies” exist. These embryopathies have been described to result in micrognathia, cleft palate, and microtia or anotia [37]. One of the affected MD calves exhibited cleft palate as observed in human retinoic acid embryopathies. Additionally, hemifacial microsomia patients manifest hypoplasia of the mandible due to decreased blood supply to the Meckel’s cartilage [38].

Finally, calves exhibited a range of severity in phenotype in the truncation of the lower jaw, presence of additional skin tags, and in one case the additional feature of a cleft palate. Variation in human syndromes affecting craniofacial development is also common. For example, the severity of hemifacial microsomia can range from moderate hypoplasia to complete absence of a portion of the jaw with other neural and muscular symptoms [4]. Similarly, in a set of infants with malformations due to RA exposure, 14% exhibited cleft palate [37]. We propose that variation in phenotype of the MD calves may be attributed to intrinsic variability in RA availability during development.

## Figures and Tables

**Table 1:** Pathologic characteristics of mandibulofacial dysostosis calves. Given is a list of the hallmark and variable characteristics observed in MD calves and indicators of which animals displayed each.

Pathologic Description	Calf 1	Calf 2	Calf 3	Calf 4	Calf 5
Bilateral bone-wrapped Meckel’s cartilage	yes	yes	yes	yes	yes
Bilateral skin tags 2–10 cm caudal to the commissure of the lips	yes	yes	yes	yes	yes
Skin tags near or below the external acoustic meatus	-	yes	yes	yes	yes
Low set and/or drooped pinnae	yes	yes	yes	yes	yes
Hypoplasia of the masseter and temporalis muscles	yes	yes	yes	yes	yes
Megastomia	yes	yes	no	no	yes
Brachgnathia inferior	yes	no	no	yes	yes
Campylognathia involving mandible and maxilla	no	no	yes	no	yes
Asymmetry of the orbits	no	no	yes	no	yes
Cleft palate	yes	no	no	no	no
Sex of calf	female	male	male	male	female

The dash (-) indicates an attribute that was not examined.

**Table 2:** Candidate variants identified from whole-genome sequence data. WGS included 3 affected calves, 10 obligate carriers, and 7 related individuals. Variants were further filtered using WGS data from additional animals.

Chr	Position (bp)	Reference	Alternative	Variant Annotation	Gene
7	15413	C	T	Intergenic	
26	10588403	T	A	Intronic	*STAMBPL1*
26	10616433	C	T	Downstream gene variant	*STAMBPL1*
26	10713132	G	A	Downstream gene variant	*FAS*
26	10794674	G	A	Intergenic	
26	10982292	TGAGAGAGGAT	TGAGAGGAT	Intronic	*LIPA*
26	14404993	T	C	Missense (p.L188P; SIFT = 0, deleterious)	*CYP26C1*
26	15898152	C	T	Upstream gene variant	*TBC1D12*

**Table 3:** Genotyping of the variant Chr26 g. 14404993T>C. Given is the count of individuals by classification for each genotype. All animals with the CC genotype were affected with MD. All parents of affected MD calves had a TC genotype.

Reporting Herd 1	TT	TC	CC	Total Animals
Founder in either maternal or paternal pedigree	95	50	0	145
Founder in both maternal and paternal pedigree	2	5	2	9
No ties to founder	91	0	0	91
Total Herd 1	188	55	2	245
**Reporting Herd 2**				
Founder in either maternal or paternal pedigree	114	35	0	149
Founder in both maternal and paternal pedigree	4	5	1	10
No ties to founder	239	0	0	239
Total Herd 2	357	40	1	398
**Other**				
Founder in either maternal or paternal pedigree	13	20	0	33
Founder in both maternal and paternal pedigree	48	37	2	87
No ties to founder	18	0	0	18
Animal is founder	0	1	0	1
Total Other Genotypes	79	58	2	139
**Total Animals Genotyped**	**624**	**153**	**5**	**782**

**Table 4:** *CYP26C1* Genotyping (Chr26 g. 14404993T>C) by source.

*CYP26C1* Genotype Source	Number of Animals
WGS for this project *	20
Hereford cattle genotyped for this project *	762
Other WGS variant data generated in our lab	101
1000 bulls [16] and American Hereford Association (WGS)	1705
Sequence Read Archive	783
**Total**	**3371**

* samples detailed in Table 3.

## Supplementary Materials

The following are available online at https://www.mdpi.com/2073-4425/11/11/1246/s1, Figure S1: Pedigree of WGS Animals, Table S1: Genotyping primers and probes, Table S2: Variants in region of homozygosity in MD affected calves on chromosome 26, Table S3: Shared homozygosity among MD calves from WGS data, Table S4: Droplet Digital PCR Proportion of Variant Alleles.
