# Supplementary material for: Mandibulofacial Dysostosis Attributed to a Recessive Mutation of CYP26C1 in Hereford Cattle
Source: Genes (Basel). 2020 Oct 22;11(11):1246. doi: 10.3390/genes11111246 (PMC7690606; doi:10.3390/genes11111246)
Supplement: Supplementary file 1 [file genes-11-01246-s001.zip › Sieck_Supplement_R1/S1 Table.docx]

**S1 Table. Genotyping primers and probes**

| Genotyping Method | Variant Location | Forward Primer | Reverse Primer | Fragment Length (bp) | Annealing Temp (C) |
| --- | --- | --- | --- | --- | --- |
| Sanger Sequencing | Chr7: 15413 | TGCATGGTCAGCCAAGTA | AATTGAAGGCCATGATAACG | 488 | 60 |
| Sanger Sequencing | Chr26: 10588399 | CGATTCGCTAAGGCTCATTC | AAGCCCTAGCACCCTAAAGC | 582 | 56 |
| Sanger Sequencing | Chr26: 10616433 | GCCCAGAGCTTTTAGCTTCC | TGGGGTTCTGGAATGGATTA | 546 | 58 |
| Sanger Sequencing | Chr26: 10982292 | TCCAATACTTTGGCCACCTC | GCAGCCACTTTTTGAAGGAG | 534 | 60 |
| Sanger Sequencing | Chr26: 14404993 | CGAGAGCAAAACCCAACACA | TCAAAGGGAAAGTCTGGGGC | 454 | 62 |
| Sanger Sequencing | Chr26: 15898152 | AACCTGACCCTGAGGGAACT | ATAATTCCAGCACCCCATGA | 520 | 62 |
|  |  |  |  |  |  |
| Genotyping Method | **Variant Location** | **Forward Primer** | **Reverse Primer** | **Wildtype Probe (HEX)** | **Variant Probe (FAM)** |
| ddPCR | Chr26: 14404993 | GCCGGTCGCTGTCTATG | GAAGAGGTTCTCCACGAACTG | CTAAAGCGCTCACCTTCCGCATGG | TAAAGCGCCCACCTTCCGCAT |
| Genotyping Method | **Variant Location** | **Primer Allele T (reference)** | **Primer Allele C (variant)** | **Common Primer** |  |
| KASP | Chr26: 14404993 | GCGGCCATGCGGAAGGTGA | CGGCCATGCGGAAGGTGG | GTCGCTGTCTATGAGGCCGCTA |  |
