# Supplementary material for: Mandibulofacial Dysostosis Attributed to a Recessive Mutation of CYP26C1 in Hereford Cattle
Source: Genes (Basel). 2020 Oct 22;11(11):1246. doi: 10.3390/genes11111246 (PMC7690606; doi:10.3390/genes11111246)
Supplement: Supplementary file 1 [file genes-11-01246-s001.zip › Sieck_Supplement_R1/S4 Table.docx]

**S4 Table. Droplet Digital PCR Proportion of Variant Alleles**

| Sample | Proportion of Variant Alleles | St Dev |
| --- | --- | --- |
| Reference Control | 0.0009 | 0.0007 |
| Variant Control | 0.9984 | 0.0009 |
| Heterozygous Control | 0.4937 | 0.0026 |
| Suspect Founder | 0.5027 | 0.0127 |
| Sire of Suspect Founder | 0.0006 | 0.0003 |
| Maternal Grandsire of Suspect Founder | 0.0006 | 0.0009 |
| Sire of Maternal Granddam of Suspect Founder | 0.0014 | 0.0006 |
