# Supplementary figures and images for: Mandibulofacial Dysostosis Attributed to a Recessive Mutation of CYP26C1 in Hereford Cattle
Source: Genes (Basel). 2020 Oct 22;11(11):1246. doi: 10.3390/genes11111246 (PMC7690606; doi:10.3390/genes11111246)

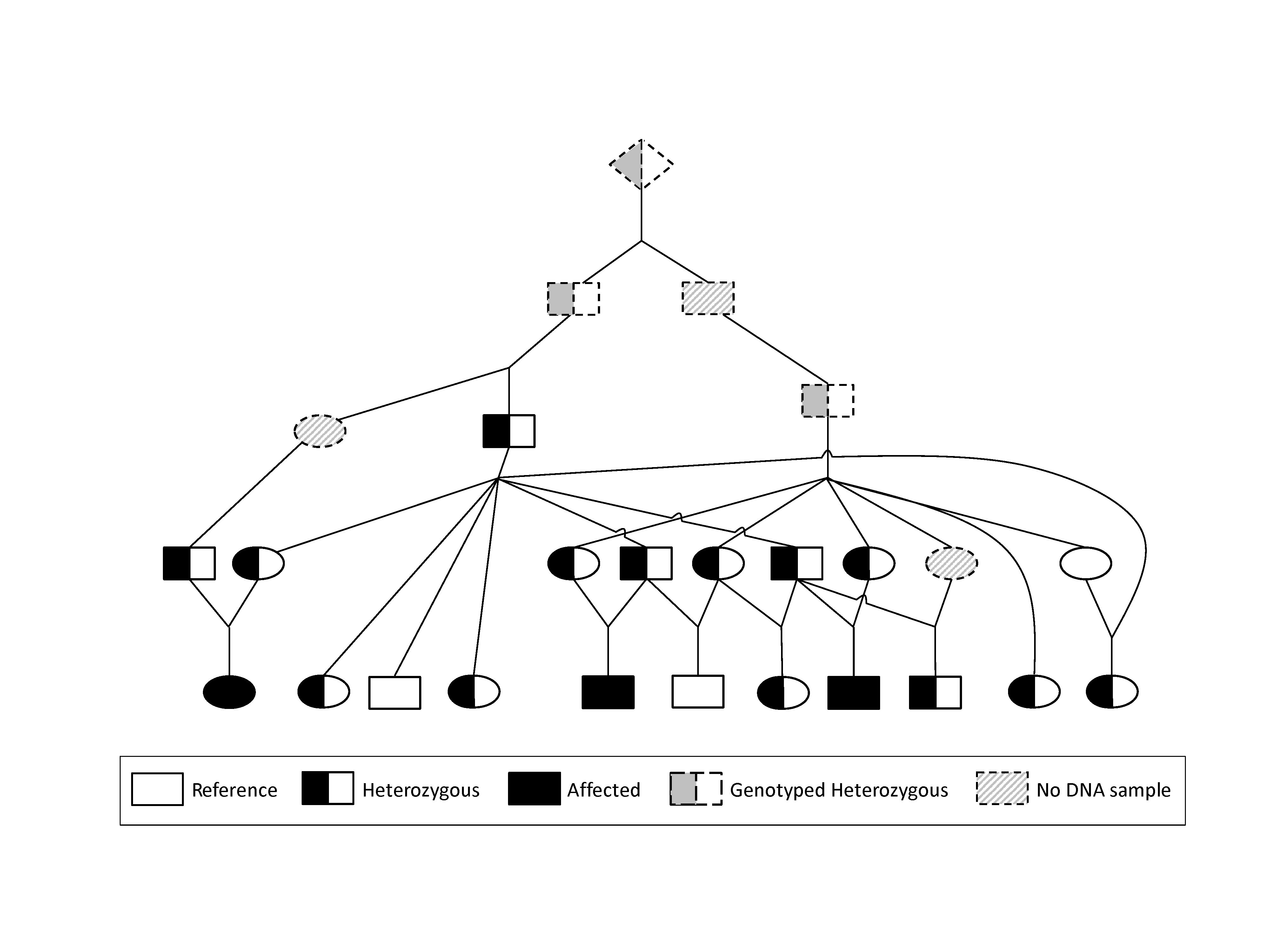

Supplement: Supplementary file 1 [file genes-11-01246-s001.zip › Sieck_Supplement_R1/S1 Fig.tif]
